# Is There a Role for Mammalian Target of Rapamycin Inhibition in Renal Failure due to Mesangioproliferative Nephrotic Syndrome?

**DOI:** 10.1155/2012/427060

**Published:** 2012-05-21

**Authors:** Hernán Trimarchi, Mariano Forrester, Fernando Lombi, Vanesa Pomeranz, Romina Iriarte, María Soledad Raña, Pablo Young

**Affiliations:** Division of Nephrology, Department of Medicine, Hospital Británico de Buenos Aires, 1280 Buenos Aires, Argentina

## Abstract

Primary glomerulonephritis stands as the third most important cause of end-stage renal disease, suggesting that appropriate treatment may not be as effective as intended to be. Moreover, proteinuria, the hallmark of glomerular damage and a prognostic marker of renal damage progression, is frequently resistant to thorough control. In addition, proteinuria may be the common end pathway in which different pathogenetic mechanisms may converge. This explains why immunosuppressive and nonimmunosuppressive approaches are partly not sufficient to halt disease progression. One of the commonest causes of primary glomerulonephritis is mesangioproliferative glomerulonephritis. Among the triggered intracellular pathways involved in mesangial cell proliferation, the mammalian target of rapamycin (mTOR) plays a critical role in cell growth, in turn regulated by many cytokines, disbalanced by the altered glomerulopathy itself. However, when inhibition of mTOR was studied in rodents and in humans with primary glomerulonephritis the results were contradictory. In light of these controversial data, we propose an explanation for these results, to dilucidate under which circumstances mTOR inhibition should be considered to treat glomerular proteinuria and finally to propose mTOR inhibitors to be prospectively assessed in clinical trials in patients with primary mesangioproliferative glomerulonephritis, for which a satisfactory standard immunosuppressive regimen is still pending.

## 1. Introduction

The universal and growing impact of chronic diseases is undoubtedly high. While there has been little attention paid to kidney disease on a public health level, the reality is that many countries hardly bear the costs of providing end-stage renal disease care through renal replacement therapy. According to the latest USRDS report, while the prevalence of diabetes has clearly increased and the prevalence of congestive heart failure has remained stable, the prevalence of chronic kidney disease appears to have declined slightly in 2009, from 15.8 percent to 15.1 percent when calculated with the MDRD-4 formula and from 14.7 percent to 14.5 percent when calculated with the CKD-EPI formula; prevalence estimates of chronic kidney disease in USA in 1988–1994 had been 12.8 and 12 percent, respectively [1]. Obviously, differences in the prevalence estimates may in part differ depending on the criteria and equations employed. Among the most frequent causes of end-stage renal disease, glomerulonephritis ranks third worldwide.

 Mesangioproliferative glomerulonephritis, mostly IgA nephropathy, is the most frequent primary glomerular disorder worldwide, and progressive mesangioproliferative nephropathy constitutes a major cause of end-stage renal disease [2, 3]. Recent long-term controlled studies assessing the prognosis have shown that the condition does not represent a benign disorder as previously mentioned [4–7]. Once renal function impairment develops, end-stage renal disease appears inevitable, often in the long term. Over 25 years of followup, about 30–50% of patients with IgAN will enter renal replacement therapy [8, 9]. Other causes of mesangial glomerulonephritis consist of pure mesangial proliferative glomerulonephritis, IgM glomerulonephritis, and C1q glomerulonephritis [10].

 Progression in mesangioproliferative glomerulonephritis generally involves increasing proteinuria, pathological renal extracellular matrix protein accumulation, cell proliferation, and inflammatory cell infiltration [11–15]. Pharmacological treatment of progressive mesangioproliferative disease is practically limited to renin-angiotensin system inhibition using angiotensin-converting enzyme inhibitors or type 1 angiotensin receptor antagonists, which serves to decelerate but is generally not capable of halting the advance of the disease [2, 13, 15]. As recently remarked by Floege and Eitner, there are few randomized controlled trials for IgA nephropathy and very rarely do patient numbers exceed 200. Consequently, most guidelines relating to IgAN are based on a low-to-very-low level of evidence and, in many cases, suggestions cannot even be offered. Thus, the majority of patients will continue to be treated based largely on opinion [16]. With respect to the other causes of mesangioproliferative glomerulonephritis, in the absence of controlled trials, it is difficult to determine the efficacy of therapy [10].

 Administration of various immunosuppressive regimes, containing steroids and cytotoxic/cytostatic drugs, is increasingly adopted. Since there is no final agreement regarding the antiproteinuric efficacy of these protocols, which also vary in different disease states, and side effects are important, the clinical efficacy of new immunosuppressors is increasingly assessed [17–19]. Among these, the mammalian target of the rapamycin (mTOR) inhibitor family, which includes sirolimus and everolimus, is frequently used immunosuppressant with proliferation signal inhibitors properties employed in nephrology [17–20]. However, serious side effects including renal injury and proteinuria have been described during treatment with these drugs, in the setting of renal allograft nephropathy, human glomerulonephritis as well as in experimental kidney disease [19, 21–23]. Despite this, mTOR inhibition has been shown to be beneficial in chronic mesangioproliferative nephropathy and to reduce proteinuria in an experimental anti-Thy1 nephritis (a chronic model of progressive mesangioproliferative nephropathy) and in focal segmental glomerulosclerosis [24].

 Aberrant proliferation of mesangial cells is a common finding in a number of diseases that can lead to end-stage renal failure. A variety of initial insults, which may be metabolic (as in diabetic nephropathy) or immunological (as in IgA disease and lupus nephritis), can cause uncontrolled mesangial cell proliferation. This in turn causes an increase in extracellular matrix deposition, ultimately leading to glomerulosclerosis, with subsequent activation of common intermediate pathways, associated with increased synthesis and a release of growth factors, cytokines, chemokines, and oxidant species, which stimulate the further proliferation of mesangial cells and finally mediating the damage of the kidney parenchyma. It is important to understand the proliferation mechanism of mesangial cell in order to determine the key regulatory molecular pathways involved in the pathological response to injury and ultimately to identify potential targets for therapeutic intervention [25].

## 2. Abnormal Cellular Mechanisms of Mesangial Proliferation in Mesangioproliferative Glomerulonephritis

**Figure 1:**
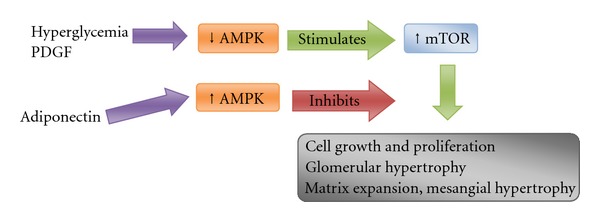
Hyperglycemia and PDGF stimulate mTOR, which in turn contribute to the nuclear translation of mRNAs necessary for cell growth and proliferation, clinically evident as hematuria, proteinuria, and glomerular filtration rate alterations.

 Platelet-derived growth factor (PDGF) has been recognized as a major mitogen and one of the most important growth factors, which mediates multiple cellular activities such as cell proliferation, hypertrophy, and extracellular matrix protein synthesis in various types of cells including mesangial cells, and plays an important role in the changes in glomerular morphology in diabetic nephropathy [25, 26] and mesangioproliferative glomerulonephritis, mainly IgAN [27] (Figure 1). In recent years, growing evidence suggests that adiponectin is also an important regulator of cell proliferation. The data about its influence on cell proliferation are conflicting. Some authors suggest that adiponectin stimulates endothelial cell growth and angiogenesis [28, 29], whereas others perceive it as a negative regulator of cell proliferation [30, 31]. Su et al. found that adiponectin inhibited PDGF-induced mesangial cell proliferation. Mechanistic insights into this phenotype suggest that adiponectin attenuates PDGF-induced phosphorylation of mTOR via AMPK activation [25]. Interestingly, in a recent study it has been reported that in subjects with mesangial cell proliferation, as IgAN and diabetic nephropathy, there exists an increase of urinary adiponectin levels, which may partly result from enhanced filtration of circulating adiponectin through the changes of glomerular permselectivity and intraglomerular hydraulic pressure [32].

## 3. Regulation of mTOR

 The mammalian target of rapamycin plays a critical role in cell differentiation, migration, and survival [33–35]. mTOR is an intracellular serine/threonine kinase and a central component of a complex signaling network that is highly conserved in evolutionary terms and expressed ubiquitously throughout the cells of the body. mTOR is a component of two major intracellular signaling complexes (mTORC1 and mTORC2), that play different roles downstream. mTORC1 is activated by growth factors and amino acids and controls cellular proliferation, promoting processes such as DNA translation, RNA transcription, ribosomal biogenesis, and cell cycle progression [35]. Inhibitors of mTOR, such as rapamycin or everolimus, bind to an intracellular cytoplasmatic receptor, the FK506-binding protein-12. The complex formed then interacts and disrupts mTOR function and leads to cell cycle arrest in the G1 phase. In addition to blocking cell proliferation, mTOR inhibitors have been found to be anti-inflammatory, antifibrotic, antitumoral, and antifungal, which underscores the involvement of mTOR signaling in a wide range of cellular functions [33, 34, 36] (Figure 1). Thus, mTOR is essential for the proliferation of mesangial cells, as is the case of mesangioproliferative glomerulonephritis. In this regard, in vitro studies with mice mesangial cells under the effect of different concentrations of rapamycin showed that a remarkably low dose (0.01 ng/mL) inhibited both proliferation and type IV collagen production. However, this dose is below that used to produce clinically immunosuppressive effects (4.5–14 ng/mL), but could also minimize other potential adverse drug effects [37].

## 4. mTOR Inhibition: Low versus High Dose

 In experimental studies, different doses of mTORi may play opposite effects on mesangial expansion. This may appear to be particularly true with respect to mesangioproliferative glomerulonephritis. In this respect, low-dose rapamycin (2.5 mg/kg^−1^/body weight^−1^ in rats) confers antiproteinuric effects in a chronic model of progressive mesangioproliferative nephropathy, that is, anti-thy1-induced glomerulosclerosis in the rat [36]. Briefly, rapamycin remarkably limits the progressive course of chronic anti-thy1 antibody-induced renal disease towards glomerulosclerosis, tubulointerstitial fibrosis, and renal insufficiency. Renoprotection by low-dose rapamycin presents beneficial effects on a number of key pathways of renal disease progression, that is, proteinuria, renal matrix protein accumulation, cell proliferation, and leukocyte infiltration [12–15, 36]. Moreover, the beneficial effects of mTOR inhibition have recently been reported in several rat models of chronic kidney disease, that is, hypertensive 5/6 nephrectomy, diabetic nephropathy, hypertrophy following unilateral nephrectomy, tubulointerstitial fibrosis due to uretheral obstruction or nephrotic syndrome, and polycystic kidney disease, but not chronic transplant glomerulopathy [38–43]. Since these experimental studies represent the vast majority of human chronic kidney disease, their findings together imply that mTOR signaling acts as a rather common key pathway in the progression of renal disease. Furthermore, these studies suggest that inhibition of mTOR might be a novel, generally effective therapeutic approach to chronic kidney disease. However, the latter option may also apply to early preventive treatment, since this represents generally the timing of mTOR inhibition in experimental rat studies. However, the outcome of mTOR inhibition in anti-thy1-induced glomerulosclerosis contrasts with the one previously reported in anti-thy1-induced acute glomerulonephritis [44, 45]. In the latter, the mTOR inhibitor everolimus before or during the early marked mesangial cell proliferation turned out to be detrimental, manifesting aggravation of proteinuria, impaired self-healing, increased uremic mortality, and persistent glomerular fibrotic changes. These effects were observed both with high- and low-dose everolimus [44]. The studies in anti-thy1 acute and chronic renal disease unanimously indicate that unaffected mTOR signaling is critical for the very early and marked mesangial cell proliferation and subsequent normal glomerular repair of acute anti-thy1 glomerulonephritis [36, 44, 45]. In the further course of the disease, inhibition of mTOR even acts beneficially and prevents chronic disease progression [36]. Daniel et al. [45] reported renoprotective effect of sirolimus in experimental mesangioproliferative glomerulonephritis. Application of everolimus as late treatment for 14 weeks attenuated proteinuria and the time course of chronic anti-Thy1 nephritis in the rat, through reduction in vascular endothelial growth factor VEGF and TGF-*β*1expression [38].

 VEGF is a potent mitogen expressed in podocytes and tubular cells that normally facilitates both glomerular and interstitial endothelial proliferation and angiogenesis [38, 46, 47]. Outlined by Schrijvers et al., the role of VEGF in normal renal physiology is essentially unknown [48]. However, VEGF and its receptors are upregulated in experimental animals and humans with type 1 and 2 diabetes and mesangioproliferative glomerulonephritis as IgAN [48–50]. Overexpression of VEGF-A leads to glomerular collapse, proteinuria, and end-stage renal disease in mice [50, 51]. In subjects with early IgAN, expression of VEGF is upregulated [52]. Some VEGF polymorphisms may be associated with the increased risk of renal progression in patients with IgA nephropathy [50]. Finally, VEGF expression is genetically regulated via the mTOR pathway [53, 54], which is in turn well known to be inhibited by both sirolimus and everolimus.

In contrast to Daniel et al. [44], Ramadan et al. have shown that either early or late low (20 mg/L) but not high doses (100 mg/L) of everolimus attenuated the loss of the slit diaphragm proteins nephrin and podocin in adriamycin-induced nephrotic syndrome (an experimental nephropathy that mimics minimal change disease), suggesting that the antiproteinuric effect of this agent at the therapeutic dose is due to preservation of nephrin and podocin. Specifically, chronic administration of everolimus at high therapeutic doses to normal rats did not induce renal injury as expressed by the lack of adverse effects on glomerular nephrin/podocin abundance. However, the authors speculate that this high-dose effect may be due to a decline of glomerular filtration rate and to the hypoalbuminemia seen in these rats [24]. 

 The efficacy of mTORi in mesangioproliferative glomerulonephritis is limited. These potential beneficial results achieved in experimental studies are now expanding towards a progressive model of human mesangioproliferative nephropathy, an important cause of end-stage kidney disease worldwide [2, 3]. However, clinical studies with sirolimus are scant and showed controversial results with other causes of glomerulonephritis. For example, while some studies reported a rapid decline in renal function with worsening proteinuria in patients with primary focal and segmental glomerulosclerosis [55, 56], others showed beneficial anti-proteinuric effects in this disease [39, 40]. However, with respect to mesangioproliferative glomerulonephritis and low-dose rapamycin, an interesting and provocative manuscript has recently been published. In this clinical study which included 25 subjects (15 on rapamycin), it has been shown that low-dose rapamycin plus angiotensin-converting enzyme inhibition (enalapril 5 mg/day) and statins (atorvastatin 10 mg/day) stabilized renal function and reduced glomerular proliferation in subjects with stage 3 chronic kidney disease measured with ^51^Cr-EDTA technique due to IgA nephropathy with proteinuria >1 g/day. At 1 year, according to Oxford classification rapamycin treatment was associated with a significant reduction of mesangial and endocapillary proliferation. Moreover, patients receiving only angiotensin-converting enzyme inhibition and statins lost 8 mL/min/1.73 m^2^, whereas those under rapamycin improved by 5 mL/min/1.73 m^2^ (*P* = 0.03). However, sclerosis, chronic biopsy lesions, and proteinuria decreased similarly in both study groups [57]. With respect to proteinuria, baseline values dropped from 2.9 ± 1.8 to 2.0 ± 0.9 g/day at one year, compared with the respective decrease in the control group from 3.7 ± 1.6 to 2.8 ± 1.4 g/day. The dose of rapamycin employed was 1 mg/day with through levels between 4 and 8 ng/mL, as it is usually used in renal transplantation. Nevertheless, authors consider this rapamycin approach as a low-dose regime, as they compare it with a previous work by Fervenza et al. in which different classes of glomerulonephritis with proteinuria >1 g/day and creatinine clearance >20 mL/min were treated with rapamycin 5 mg/day to seek through levels between 7 and 10 ng/mL [23]. However, in this work renal function was measured with Cockcroft-Gault equation, only 5 of the 11 subjects included had IgA nephropathy, 6 developed acute renal failure presumably due to not-adjusted rapamycin levels according to renal function, and no data regarding initial or final proteinuria are offered [23]. Therefore, we believe both studies are not comparable, and the human “low-dose” mTORi for mesangioproliferative is still to be determined.

 Finally, mTORi are not recommended in the transplanted patient when proteinuria exceeds approximately 800 mg/day. However, proteinuric mechanisms in this setting are not related to mesangial proliferation, which is the main culprit in mesangioproliferative glomerulonephritis. In addition, low dose of everolimus or rapamycin in human is to be determined.

 We believe that rapamycin and everolimus must not be considered indistinctly as therapeutic alternatives in glomerular diseases. Human doses have not been established, and randomized trials in humans are required before recommendations are made. Whether preferentially early versus late, low-versus-high doses of mTORi effects on proteinuria in mesangioproliferative GNs are due to VEGF reduced expression, to podocin-nephrin protection, and/or to interventions in the adiponectin-PDGF binomium, among many other possibilities, is a novel, interesting, and a promising field of research to assess treatment of acute mesangioproliferative glomerulonephritis, for which no specific immunosuppressive standardized therapy exists.
